# Digital leadership and exploratory innovation: From the dual perspectives of strategic orientation and organizational culture

**DOI:** 10.3389/fpsyg.2022.902693

**Published:** 2022-09-13

**Authors:** Tiandong Wang, Xiaoyue Lin, Fan Sheng

**Affiliations:** ^1^School of Economics and Management, Harbin Engineering University, Harbin, China; ^2^School of Business and Management, Jilin University, Changchun, Jilin Province, China

**Keywords:** digital leadership, digital entrepreneurship orientation, digital organizational culture, big data analytics capabilities, exploratory innovation

## Abstract

The literature on leadership is increasingly supporting the power of digital leadership in promoting corporate innovation. In spite of this, digital leadership is a noticeable omission from the literature. As such, in this study, we developed a model based on a resource-based view and social information processing theory to examine the roles of digital entrepreneurial orientation and digital organizational culture in the relationship between digital leadership and exploratory innovation. We examined the moderating role of big data analytics capabilities according to a resource-based view and dynamic capability theory. Using a time-lagged survey data of 401 followers and 88 leaders, the results show that (a) digital leadership has a positive impact on exploratory innovation; (b) digital entrepreneurial orientation and digital organizational culture mediate the positive relationship between digital leadership and exploratory innovation; and (c) and mediating effect is positive moderated by big data analytics capabilities. Thus, in this study we are not only responding to the call to strengthen digitalization research in organizations but also further deepening our understanding of the path from digital leadership to exploratory innovation. These findings have theoretical implications for the literature on leadership and managerial implications for practitioners.

## Introduction

Due to the disruptive nature of digital technologies (Karimi and Walterm, 2015), the global digital landscape is changing at an exponential rate. Enterprises generally face the challenge of digitalization in a volatile, uncertain, complex, and ambiguous (VUCA) environment. In reality, the success rate of the digital transformation of enterprises is less than 30%, and the support and leadership of middle and senior managers are central to achieve digital transformation (Oberer and Erkollar, 2018; Mihardjo et al., 2019a). Digitalization has led to changes in the nature and performance of leadership, including facilitating instant access to information and expansive datasets (Mazzei and Noble, 2017); creating new communication principles (Bennis, 2013); making changes in leadership education (Sia et al., 2016); leading to decision making that is increasingly based on the intelligent analysis of big data; and creating new leadership positions such as CTO, virtual teams, etc. (Schwarzmüller et al., 2018; Nadkarni and Prügl, 2021). These changes require leaders to apply new, dynamic, and continuous learning leadership—that is, digital leadership (Kane et al., 2019)—to lead enterprises to achieve digital strategic goals. Digital leadership emphasizes five key capabilities for leaders: creativity, thinking and inquisition, curiosity, deep knowledge, and global vision and collaboration (Zhu, 2015).

Although corporate practice and the consulting industry have transformed leadership in the digital world into digital leadership or leadership 2.0 (Hesse, 2018), researcher enthusiasm for this important phenomenon in academia is only just being ignited. The body of literature that recognizes the importance of digital leadership is growing. Overall, studies on digital leadership outline its origins, concepts, and characteristics, superficially covering topics related to digitalization, the Internet, systems, and organizations, and preliminarily verifying the relationship between digital leadership and dynamic capability (Mihardjo et al., 2019a), innovation management (Wasono and Furinto, 2018), strategic alliances, market orientation (Mihardjo et al., 2019b), and other variables. Despite the awareness of the important role of digital leadership in digital transformation and innovation, the results in the literature are relatively limited and high-quality research is lacking.

This paper attempts to address several research gaps in digital leadership research: (1) Digitalization is a rather young phenomenon and research area, with the existing literature focusing on internal process and strategy. There are very few studies discussing the digitalization of organizational management, and even less is known about the role of digital leadership in the digitalization process of companies (Gfrerer et al., 2020; Philip, 2021). Thus, discussion on the outcome variables of digital leadership is inadequate. Our study investigates the role of a new type of leadership, digital leadership, under the digitalization context, and finds that digital leadership has a significant effect on corporate innovation, culture cultivation, and orientation building. It enriches the research on the results of digital leadership in organizations and the role that “people” can play in the digitalization process. (2) Although Mihardjo et al. (2019a) have confirmed that digital leadership contributes to corporate innovation management, the mechanisms and pathways have not been explored. It is necessary to discuss these in the context of specific perspectives. Meanwhile, in the digital era characterized by ever-shortening market and technology iteration cycles, the survival and development of companies rely more on disruptive exploratory innovation rather than corporate innovation in a general sense. Yet, the empirical model of digital leadership and exploratory innovation has not been constructed, and consequently, the related variables of the relationship are unclear. To this end, we try to construct an empirical model of digital leadership and exploratory innovation and find that digital entrepreneurial orientation and digital organizational culture play mediating roles. (3) Prior research has asserted that certain leadership styles, for instance, transformational leadership (Chen et al., 2019), inclusive leadership (Gong et al., 2021), participative leadership (Chang et al., 2019), and distributed leadership (Berraies et al., 2021), can positively influence exploratory innovation. Despite these significant contributions, critical omissions in the literature need to be addressed to theoretically and empirically advance this line of research. Digital leadership is among those breakthroughs. Focusing on the important role of digital leadership in corporate innovation, this paper expands the leadership style variables that affect exploratory innovation. (4) Big data analysis capabilities are a variable that has received a lot of attention recently and is considered to be the vital capabilities for enterprises to realize digitalization (Mikalef et al., 2017). Important tasks for digital leaders include driving businesses to leverage insights from big data analysis to improve data availability, thereby preparing for the next move to create value (Zhu, 2015). At the same time, the role of digital leadership requires big data analysis as a support and tool, which determines that big data analysis capabilities play a significant role in the impact of digital leadership on exploratory innovation. The specific role of big data analysis capabilities remains to be studied. Accordingly, we incorporate big data analysis capabilities into the model and discuss its moderating effect.

The goal of this study is to examine the pathways and mechanisms by which digital leadership influences exploratory innovation. To this end, we referred to Oberer and Erkollar (2018) and Nadkarni and Prügl (2021) in their studies of digitalization by considering the human actors (organizational management) and material innovation/technology (business management) aspects. A moderated mediation model is constructed. The findings reveal that digital leadership mediates the relationship between digital leadership and exploratory innovation through digital entrepreneurial orientation and digital organizational culture, and that big data analytics capabilities strengthen the mediator effect of digital entrepreneurial orientation and exploratory innovation. Throughout the study, resource-based view (RBV) and social information processing theory (SIP) contributed to generating fresh insight into this issue from the dual perspectives of strategic orientation and organizational culture. The former is motivated by rapidly changing market demand, whereas the latter is driven by employee participation and motivation. Specifically, RBV demonstrates the logic provided by digital leadership to companies that is necessary to form a digital entrepreneurial orientation and fosters exploratory innovation by actively trying and pursuing the new (Kollmann and Stöckmann, 2014). Simultaneously, from the perspective of SIP, organizational members adjust their attitudes and behaviors based on the cues and information released by digital leadership regarding organizational support and desired behaviors. In turn, a common mindset and cultural attitude gradually form within the enterprise *via* social interaction processes. Digital organizational culture enables enterprises to meet contemporary challenges; inspires enterprise members to form digital vision, thinking, and subjective initiative; and then creates a substantial push for exploratory innovation (Schiuma et al., 2021). Digital entrepreneurial orientation and digital organizational culture are considered as mediating variables between digital leadership and exploratory innovation. Furthermore, RBV and dynamic capability theory originally emerged to explain how big data analytics capabilities convert ownership or control of tangible resources, intangible resources, and human skills and knowledge into insights, positively impacting firms to more proactively and rapidly respond to upcoming business opportunities, which further promotes exploratory innovation (Akter et al., 2016; Mikalef et al., 2017). Therefore, big data analytics capabilities are regarded as a moderating variable between the above two sets of relationships. Our study benefits understanding of the path of digital leadership and exploratory innovation.

## Theory and hypotheses

### Digital leadership

The study of digital leadership is based on Hambrick and Mason’s (1984) upper echelon theory (Wasono and Furinto, 2018). The central argument of the upper echelon theory is that leaders’ experience, values, and personalities influence their choices (Hambrick, 2007) and, through these choices, influence organizational performance (Hambrick and Mason, 1984). The theory has become a catalyst for examining how the characteristics and experiences of leaders shape their perceptions, choices, and actions in ways that ultimately affect various corporate outcomes (Neely et al., 2020), and it has been well applied to leadership styles such as entrepreneurial leadership (Mehmood et al., 2021) and empowering leadership (Ou et al., 2014). Having a digital leadership style gives leaders unique characteristics and performance that influence a leader’s behavior and decisions, thus having an impact on firm-level variables. In recent years, interest in digital leadership has been increasing, but no consistent concept has yet been formed. The two main types of understanding of digital leadership are represented: first, that of Waal et al. (2016), who consider digital leadership as a combination of transformation leadership and digital technologies, i.e., the ability of digital leaders to identify and realize opportunities to create value through the effective, efficient, and acceptable use of digital technologies; the second is represented by Mihardjo et al. (2019a,b), who claim that digital leadership consists of an integration of culture and digital competencies in using digital technologies as part of the leadership style to generate value for the firm. Sawy et al. (2016) added a business ecosystem element to this definition, arguing that digital leadership is the capacity of performing the right activities to achieve strategic success of digitalization for the enterprise and its business ecosystem. Oberer and Erkollar (2018) developed a two-dimensional 4.0 leadership style matrix in the age of Industry 4.0, where the x-axis describes the individual’s capability and focuses on technology and innovation orientation, and the y-axis describes the concern for people demonstrated by the leader. The four quadrants correspond to the four leadership styles: TL (4.0 technology leader), DL (4.0 digital leader), EL (4.0 social leader), and FL (4.0 freshmen leader).

The literature provides rich discussions on the leadership characteristics or capabilities of digital leadership, with the study of Zhu (2015) being the most representative and influential. Zhu (2015) defined digital leadership as consisting of five characteristics: (1) as thought leaders, digital leaders must have deep knowledge and depth of understanding in learning and change; (2) as creative leaders, digital leaders must have creativity and an innovation mindset that can formulate the idea for the future into a reality in business; (3) as global visionary leaders, digital leaders can provide direction and become an orchestrator of digital business transformation; (4) as inquisitive leaders, digital leaders must have learning capability and the ability to implement that learning and digital capability; (5) as profound leaders, digital leaders have a wealth of knowledge and in-depth understanding to apply in decision making. The above characteristics focus only on the leader’s skills. Waal et al. (2016) described digital leadership as a combination of skills and characteristics that influence and induce other people to perform effectively, and the key is to communicate digital capabilities (Schiuma et al., 2021). Imran et al. (2020) further investigated the fundamental skills distinguishing digital leaders and identified five critical digital leaders’ competencies: digital vision, digital knowledge, failing fast, empowerment, and managing diverse teams. He expanded the study of digital leadership characteristics by addressing them from two perspectives: leadership and the interaction between leaders and followers, which is highly illuminating. In addition, Bennis (2013) stated that digital leaders must have adaptive capacity, coupled with resilience and openness to the new. McCarthy et al. (2021) focused on leadership in digital transformation; they used the term “digital transformation leadership” to identify eight digital transformation leadership characteristics: digital strategist, digital culturalist, digital architect, customer centrist, organizational agility, data advocate, business process optimizer, and digital workplace landscaper.

### Exploratory innovation

Within innovation, innovation management is divided into two major categories: exploratory and incremental innovation. Exploratory innovation refers to the acquisition and application of new knowledge to develop completely new products or services, processes, and models for new customers or emerging markets (Sheng and Chien, 2016; Wasono and Furinto, 2018). It reflects a high degree of novelty that changes the overall order of things and is seen as the skill used to gain a competitive advantage (Sheng and Chien, 2016). Incremental innovation involves enhancing existing knowledge to improve existing products or services, processes, and models (Sheng and Chien, 2016; Nguyen et al., 2018), with low novelty, low cost, and low risk being associated with small changes in things (Lei et al., 2019). Compared to incremental innovation, exploratory innovation is more complex, has a higher resource base, cost, and risk, but is also more capable of capturing new market opportunities and expanding its advantages in the digital era. As a result, exploratory innovation is more important for the survival and development of firms (Lumpkin and Dess, 2001). Here, we aim to provide a versatile platform for a broader research program on exploratory innovation.

### Digital leadership and exploratory innovation

Digital leadership positively impacts exploratory innovation (Wasono and Furinto, 2018). Kohli and Johnson (2011) reported that the role of digital leaders is central to driving rapid decision-making and change when implementing digital transformation and innovation. In this new situation, digital leaders face different requirements. Company strategy and IT strategy, which synergistically comprise a digital business strategy, must be combined. Four areas need to be defined within a digital business strategy: the scope, scale, and speed of digital business strategy and the sources of value creation (Bharadwaj et al., 2013). Digital leaders who are creative and have digital visionary fully understand the importance of exploratory innovation. They develop exploratory innovation as an essential element of digital business strategy and strategically support it to drive enterprise-wide implementation of exploratory innovation in a top-down manner. In addition, digital leaders encourage the effective and efficient use of digital tools across all departments (Borowska, 2019), which involves promoting the levels of automation and intelligence in internal operations (e.g., office suite software) to improve operational efficiency and management quality, freeing members from mechanical work to focus on more complex and cognitive work. This reshapes the structure of social networks with suppliers and customers, thereby increasing opportunities to explore new methods of creating value, leading to profound changes in products and services, organizational frameworks, and business models. Given this background, we constructed Hypothesis 1.

*Hypothesis 1*: Digital leadership has a positive impact on exploratory innovation.

### Digital entrepreneurial orientation as a mediator

In the digital age of changing environmental conditions, entrepreneurial orientation is no longer exclusive to new enterprises. The need for incumbent enterprises to implement entrepreneurial activities and establish entrepreneurial orientation to create additional value is widespread. Entrepreneurial orientation describes the continuous drive of firms to deal with business uncertainty and continuously seek new opportunities in entrepreneurial activities (Wiklund and Shepherd, 2005), which is an aspect of the category of strategic orientation of firms that reflects entrepreneurship and the willingness to engage in entrepreneurial behavior (Kollmann and Stöckmann, 2014). Entrepreneurial orientation is a combined representation of innovativeness, proactiveness, and risk taking (Miller, 1983; Covin and Slevin, 1991). Innovativeness refers to the willingness and ability to engage in innovative ideas, novelty, experimentation, and innovation processes. Proactiveness refers to anticipating future needs and trends and acting on them, thus creating a first-mover advantage over competitors. Risk taking refers to the propensity to implement high-risk projects and the willingness to advance boldly without completely knowing potential consequences. Moreover, drawing on the idea of green entrepreneurial orientation, we propose that digital entrepreneurial orientation is a derivative concept of entrepreneurial orientation in the digital domain, which is the continuous drive of firms to actively use digital technologies in digital entrepreneurial activities to deal with uncertainty in business and to constantly seek new opportunities. In contrast to entrepreneurial orientation, digital entrepreneurial orientation includes market responsiveness agility in addition to innovativeness, proactiveness, and risk taking. This is because digital products or services are changing at a much higher rate, and they intermittently and cyclically vary. An agile and lean approach is required to respond and adapt to customer needs promptly, improving the efficiency and responsiveness of new products and/or services development.

Digital leadership influences exploratory innovation through digital entrepreneurial orientation. RBV is widely considered to be one of the most prominent and powerful theories explaining how firms achieve and sustain competitive advantage through the resources they own or control (Barney, 2001). One of the most prominent and powerful theories explains how firms achieve and sustain competitive advantage through the resources they own or control (Barney, 2001). RBV researchers have long theorized that resources as rare, inimitable, and nonsubstitutable firm-specific assets that are essential to gain competitive advantage (Barney, 1991). Strategic orientation plays an important role in the resource value transformation process. RBV explains the motivational effect of entrepreneurial orientation by requiring leaders to provide innovative, proactive, risk taking, and agile resources and to draw knowledge from the external environment to stimulate innovative behavior. Lumpkin and Dess (2001) found that entrepreneurial orientation is associated with entirely new resource package creation (Kollmann and Stöckmann, 2014). Liu and Lee (2019) uphold that entrepreneurial orientation is an intangible resource for implementing strategies and increasing innovation capabilities, which is difficult for competitors to detect, observe, and imitate. Digital entrepreneurial orientation is related to leadership attitudes toward change, innovation, risk taking, and competition with other firms. Digital leaders are digitally literate and adaptable and take a positive view of change to promptly master and apply the most cutting-edge technologies and to activate all internal and external resources (including R&D capital investment, external professional analysts, etc.) to ensure companies stay innovative. Digital leaders also have forward-looking perspectives, clear vision, sound strategy, and foresight that lead to companies making quick decisions without having complete information (Schiuma et al., 2021). These practices can avoid the loss of opportunities and support companies in demonstrating a certain level of risk taking and proactiveness. Furthermore, digital leaders possess market and trend knowledge, business acumen, problem-solving skills, and the ability to fail and learn quickly, which support the agile development behavior of enterprises in obtaining or building unique strategic resources.

Isichei et al. (2020) stated that a positive link exists between digital entrepreneurial orientation and firm innovation. Innovativeness orients the firm toward embracing experimentation, technological leadership, and research and development (R&D) to generate novel products, services, and processes (Bhuian et al., 2005). These qualities can enable firms to deploy new product–market portfolios and adopt advanced technologies, processes, and methods in the creation of goods and services (Kollmann and Stöckmann, 2014). Pioneering drives firms to identify and exploit opportunities in their environment toward taking initiatives, pursuing opportunities, and shaping future needs, helping them to break through traditional management and behavioral inertia (Tang et al., 2012), thereby creating new resources cheaper and faster than competitors (Lumpkin and Dess, 2001) and gaining market and industry precedence. Risk taking is a high-resource-absorbing orientation that increases the speed of decision making and allows firms to seize opportunities within a short window of time, so firms engaging in exploratory innovation must tend toward accepting financial and business risks (Kollmann and Stöckmann, 2014). Agility represents firms’ ability to capture user needs clearly and quickly, allocate resources flexibly, and drive more efficient use of resources to redefine new markets, new user relationships, and new business models so that innovative products and services respond to and lead the potential needs of users. Hence, we constructed the following hypothesis:

*Hypothesis 2:* Digital entrepreneurial orientation mediates the positive relationship between digital leadership and exploratory innovation.

### Digital organizational culture as a mediator

Since the concept of organizational culture was popularized in the 1980s, this topic has received considerable attention from both management scholars and practitioners. Organizational culture is thought to be a source of sustained competitive advantage, a key factor in organizational effectiveness and critical to the success of projects involving organizational change. Martínez-Caro et al. (2020) argued that in the era of the digital workforce, organizational culture must extend to include its digital workplace practices, defining digital organizational culture as a set of shared assumptions and understanding about organization functioning in a digital context. Specifically, digital organizational culture involves involvement, consistency, adaptability, and mission (Denison et al., 2014). Organizational culture has been generally thought of as a long-standing and relatively stable feature that may be difficult to change on a large scale but can evolve by finding new digital approaches to reinforce it in formal and informal ways, providing the foundation needed by organizational members to recognize change and implement adaptations within the digital context (Costanza et al., 2016). The core of digital organizational culture is a combination of artifacts. According to Duerr et al. (2018), adapting organizational culture to the digital environment involves the following: (1) Due to novel methods of internal collaboration (e.g., physical and virtual collaboration and dual structures) and external collaboration (e.g., platforms with competitors and partners in addition to customer integration), artifacts are becoming evident in the changing structure of digital companies. (2) Values are the digital goals and norms that are seen as critical to the new organizational culture. (3) The underlying assumptions of companies operating in the digital age relate to the need to integrate IT into innovation or equal distribution of power, which empowers employees by integrating their ideas into the digital strategy. Although technological and economic changes produced by digitalization have received considerable attention, digital organizational culture has received little focus as an essential driver of digital transformation and innovation. Researchers in this area need to analyze how digital organizational culture may serve as a facilitator of companies’ digital transformation.

Digital leadership influences exploratory innovation through digital organizational culture. SIP suggests that employees’ psychological perceptions and behavioral decisions begin with the social information available in the work situation and the processing, processing, and response to that information, following the “social information–perception–behavior–output” response paradigm (Salancik and Pfeffer, 1978). Leaders are an important source of information for employees, and employees determine which behaviors will be approved by their leaders based on their leadership style (Lu et al., 2018). The messages released by leaders in the workplace can influence not only individual attitudes and behaviors but also the collective culture and climate. This is because workers receive messages from leaders as well as from other members within the team. After diverse team members communicate and react to the messages delivered by the leader to reach a consensus, the corresponding organizational culture is formed. Digital leaders focus on interaction and communication with employees and are thus able to deliver rich information and cues to employees in interaction, thus influencing the way employees interact and behave in the team to shape and develop organizational culture, which is a key catalyst for developing a change-oriented digital organizational culture (Schiuma et al., 2021). Wasono and Furinto (2018) further suggested that the output of digital leadership at the organizational level depends on the influence of leaders’ decisions on participants’ perceptions. As a key clue offered by the context, digital leadership conveys the message that participation in digital transformation and innovation is beneficial to corporate members. Members develop a shared perception that exploratory innovation is the appropriate behavior through peer-to-peer communication and superior–subordinate communication. Digital technologies enable digital leaders to reach more employees and a wider audience than ever before. Digital leaders tend to adopt new communication and interaction methods to disseminate the digital mission, vision, and values to members. They use stories to create scenarios and visions, codify tacit knowledge, explain ideas, smooth the implementation of change, overcome mental barriers people build against new knowledge (Schiuma et al., 2021), and even use social media and technological tools (Cortellazzo et al., 2019). Positive support from leaders enhances a unified vision and goals across the enterprise. Additionally, digital leaders lead in a more participatory manner. They are more likely to care about members’ emotions and needs, trust and respect their opinions, and support them to develop digital knowledge and skills. These initiatives prompt employees to align with the corporate strategic vision, triggering the intrinsic motivation to contribute to the enterprise (Schwarzmüller et al., 2018), thereby forming a unified digital organizational culture.

Digital organizational culture has a facilitating effect on exploratory innovation (Chandler et al., 2000). First, digital organizational culture sets the tone for digital change and influences the acceptance of new technologies and ideas. Because digital technologies do not create value by themselves, value can only be developed when the digital organizational culture matches the visions embedded in digital technologies (Büschgens et al., 2013). The digital organizational culture also ensures that innovative activities are recognized by leaders and followers and inspires knowledge sharing and creative activities among members (Martínez-Caro et al., 2020). The generation of new and valuable ideas or the execution of work in novel and appropriate ways is encouraged (McLean, 2005), which stimulates exploratory innovation. Thus, we propose the following hypothesis:

*Hypothesis 3*: Digital organizational culture mediates the positive relationship between digital leadership and exploratory innovation.

### Big data analytics capabilities as a moderator

Big data analytics is defined as a holistic approach to managing, processing, and analyzing the 7 V data-related dimensions (Wamba et al., 2017), including not only the subject matter, i.e., data, but also tools, infrastructure, visualization, and methods of presenting insights (Mikalef et al., 2017). Nowadays, research has been identified as the next frontier after innovation, competition, and productivity (Mikalef et al., 2019). According to dynamic capability theory, big data resources do not necessarily produce growth in business value, so enterprises need to integrate, build, and reallocate their resources and capabilities under changing conditions (Teece et al., 1997; Su et al., 2021). However, big data analysis can only play a role when the technology cooperates with human skills to convert raw resources into high-end capabilities (Wamba et al., 2017; Gupta et al., 2020). Thus, the concept of big data analytics capabilities was formally developed (Mikalef et al., 2017). At present, no consensus exists on the definition of big data analytics capabilities (Su et al., 2021). Some scholars view big data analytics capabilities as abilities to make decisions related to organizational operation strategies depending on big data. Others broadly consider big data analytics capabilities in referencing a company’s proficiency in leveraging big data to gain strategic and operational insight (Mikalef et al., 2017). These concepts are complementary and form the cognitive logic of “data–insights–behaviors–value” (Su et al., 2021). The dimensions and measurements of big data analytics capabilities are also not unified and mainly include two factions. The first is the RBV, such as that of Grant (1991), Gupta and George (2016) and Mikalef et al. (2017). They proposed that big data analytics capabilities are divided into three main categories: tangible resources (e.g., infrastructure, IS, and data), intangible resources (e.g., data-driven culture, governance, and social IT/business alignment), and human skills and knowledge (e.g., data analytics knowledge and managerial skills). The second, the competency-based view, splits big data analytics capabilities into multiple subcapabilities. For example, Akter et al. (2016) demonstrated big data analytics capabilities as a hierarchical model that consists of three primary dimensions (i.e., management, technology, and talent capability). In general, the research on big data analytics capabilities mainly focuses on the definition, dimensions, antecedents, results, and operating mechanisms. Comprehensive knowledge is lacking about how big data analytics capabilities can be leveraged and through what mechanisms its value can be created (Su et al., 2021).

According to the basic philosophy of RBV, an organization is viewed as a set of valuable tangible and intangible resources that can be combined to generate competitive advantage (Peteraf, 1993). A characteristic of resources is that they do not generate any business value on their own but require that action be taken for their strategic exploitation. The resources a firm owns or controls are critical to determining what types of capabilities a firm can develop and the value of those capabilities (Wu, 2007). Big data analytics capabilities can be purposefully built by focusing on the complex interactions between a firm’s intangible resources, tangible resources, human skills and knowledge, and competencies and are therefore more complex and harder to imitate than core resources (Grant, 1996). Dynamic capability theory further suggests that big data analytics capabilities enable firms to integrate, construct, and reconfigure their resources and capabilities under changing conditions (Teece et al., 1997). Akter et al. (2016) argued that big data analytics capability is a dynamic capability that is transformed from a firm’s internal resources under complex and turbulent conditions. Using this capability, which cannot be easily acquired, firms can achieve a higher level of organizational sustainability and outcompete others (Gupta et al., 2020). Companies that are leaders in the adoption of big data analytics capabilities are more likely to produce new products and services (Su et al., 2021). Big data analytics capabilities are distinguished from other analytics capabilities in that they enable the processing of unstructured and varied data sources in much shorter cycle times (Chen et al., 2012); this processing level helps to increase the speed, effectiveness, and efficiency of insight generation. With digital entrepreneurship orientation, firms are innovative, proactive, risk taking, and agile. Big data analytics capabilities enable firms to reposition themselves in a changing business environment, generate valuable information from a wide range of external sources, and use human skills to transform knowledge and skills into actual innovation actions (Su et al., 2021). This allows companies to identify and respond more proactively and rapidly to new or even upcoming business opportunities under highly complex and rapid conditions, break through bottlenecks in operations and processes, and develop predictive models for future events, driving companies to recognize gaps or areas of ignorance and take action to adjust innovation plans (Erevelles et al., 2016; Mikalef et al., 2019). These companies are thus more likely to develop entirely new products, technologies, and production processes with higher levels of innovation and difficulty (Su et al., 2021). From the above discussion, we hypothesized the following:

*Hypothesis 4*: Big data analytics capabilities positively moderate the relationship between digital entrepreneurial orientation and exploratory innovation.

In companies with digital organizational culture, members autonomously participate in digital activities. Higher levels of big data analytics capabilities allow members to understand internal operational conditions, supply chain processes, employee performance, and consumer behavior patterns (Rialti et al., 2018), creating an understanding of analytics results. The major contribution of big data analytics capabilities is that they enable companies better to make more rational and informed decisions, which are subject to less bias and based on empirical evidence (Mikalef et al., 2019). Big data analytics capabilities also facilitate the collaboration of decentralized organizational units, alleviate the pressure to innovate in individual departments, allow for improved data-driven decision making, and innovative ways to organize, learn, and innovate, resulting in fully exploiting exploratory innovation potential embedded in heterogeneous knowledge from different sources (Ferraris et al., 2019). We therefore hypothesized the following:

*Hypothesis 5*: Big data analytics capabilities positively moderate the relationship between digital organizational culture and exploratory innovation.

### A moderated mediation model

Based on the above analysis, it can be considered that big data analysis capabilities play the moderating role in the relationship of “digital leadership-digital entrepreneurial orientation, digital organizational culture-exploratory innovation,” that is, the mediating role of digital entrepreneurial orientation and digital organizational culture is affected by big data analysis capabilities. We therefore hypothesized the following:

*Hypothesis 6*: The mediation effect of digital entrepreneurial orientation between digital leadership and exploratory innovation would be moderated by the level of big data analytics capabilities, such that the indirect effect of digital entrepreneurial orientation would be strong for those companies have a high level of big data analytics capabilities.

*Hypothesis 7*: The mediation effect of digital organizational culture between digital leadership and exploratory innovation would be moderated by the level of big data analytics capabilities, such that the indirect effect of digital organizational culture would be strong for those companies have a high level of big data analytics capabilities.

**Figure 1:**
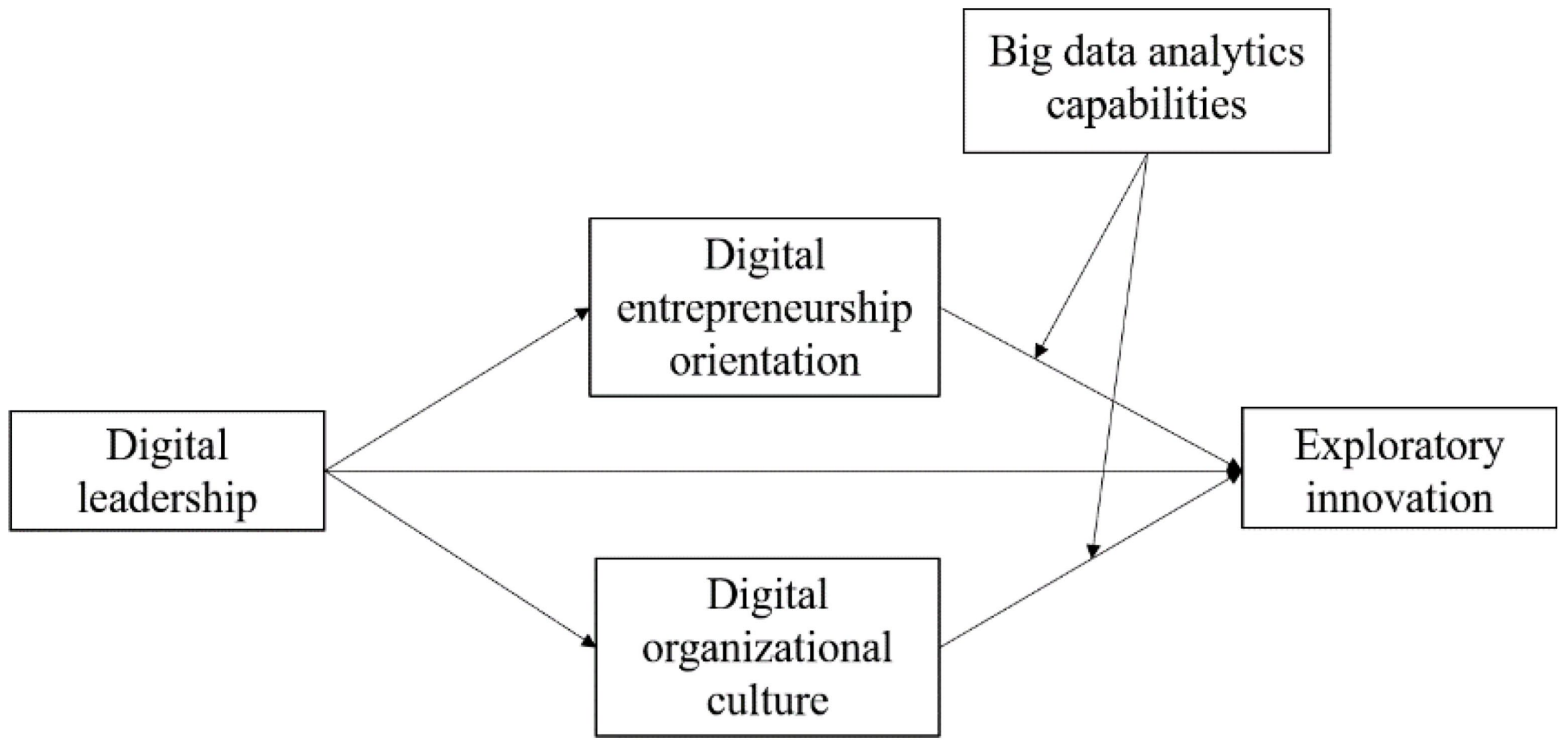
The hypothesized model.

The hypothesized theoretical model is presented in Figure 1.

### Impact pathway map

**Figure 2:**
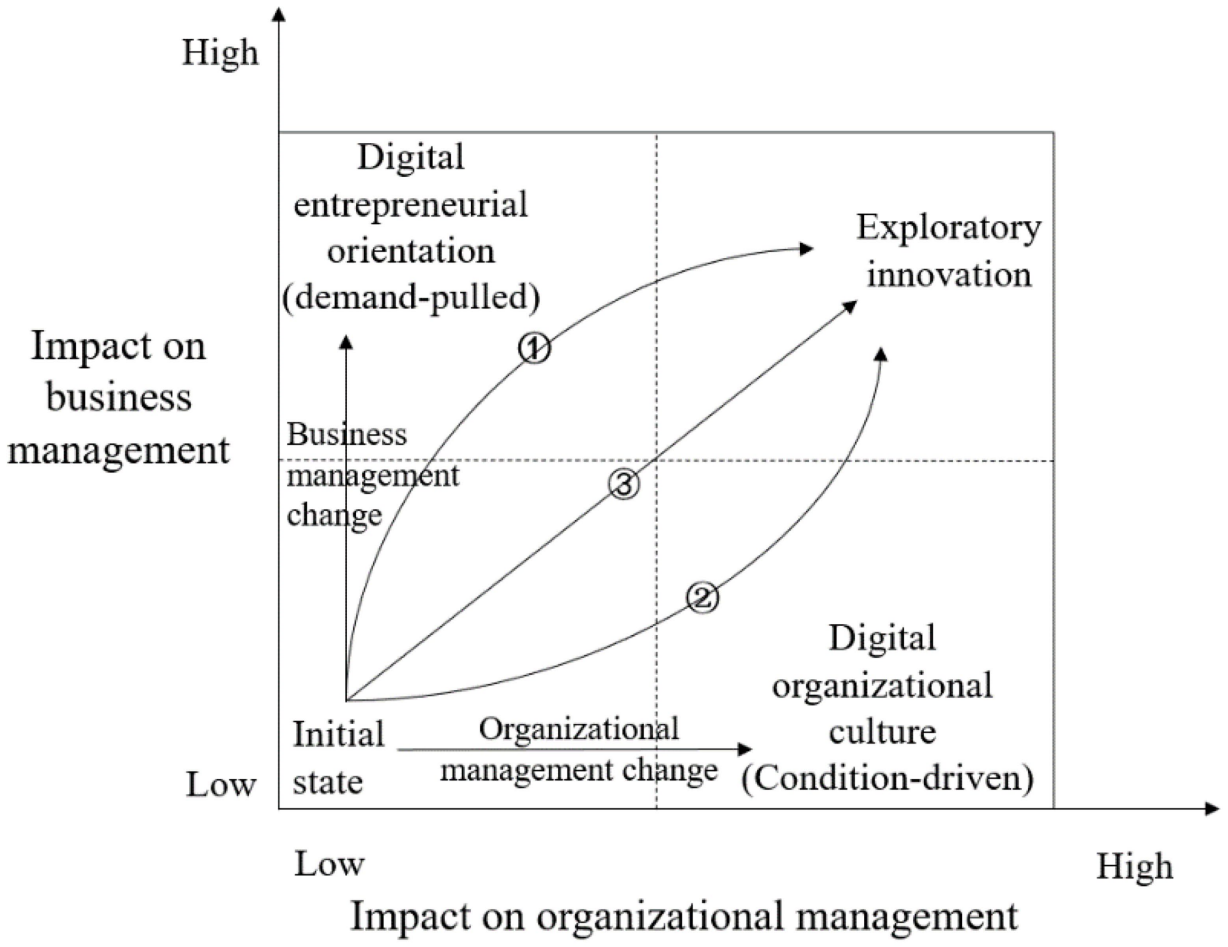
Path diagram of the impact of digital leadership to exploratory innovation. Note: ① business management route; ② organizational management route; ③ equal emphasis on management and organization.

The impact pathways research is inspired by Oberer and Erkollar (2018) and Nadkarni and Prügl (2021), who stated that digital leadership impacts both human actors (organizational management) and the material innovation/technology (business management). Built on SIP and RBV, we suggest that exploratory innovation must rethink the strategic orientation of a company in business management as well as organizational culture in organizational management. Responding to external markets and internal people are the vital tasks of digital leaders. So, digital leaders must focus on building an digital entrepreneurial orientation with active exploration and risk taking in business management. Digital organizational culture supports change, enabling more concentration on effectively achieving innovative breakthroughs (Baker et al., 2015). Therefore, we used the impact on organizational management as the x-axis and the impact on business management as the y-axis to establish a path diagram of the impact of digital leadership on exploratory innovation, as shown in Figure 2. Digital leadership influences exploratory innovation *via* three routes: First, digital leaders steer organizational management changes to build a digital organizational culture, which is driven by internal conditions to mobilize members’ participation and creativity, forming a positive interaction between people and innovation and then promoting exploratory innovation behaviors. Second, digital leaders conduct business management remodeling to establish the digital entrepreneurial orientation, which is driven by changing market demands, leading companies to enhance exploratory innovation activities earlier and faster. Third, digital leaders transform both organizational management and business management. Digitalization of products and digitalization of actors are given equal importance. Exploratory innovation behaviors are reinforced by the combined force of digital entrepreneurial orientation and digital organizational culture.

## Materials and methods

### Samples and procedures

Before the formal survey of this study, a pretest was conducted to revise and initially test the translated scale. First, three experts in related fields and five PhD students were invited to translate and correct the scale from English to Chinese, and semi-structured interviews were conducted with five entrepreneurs to revise the questionnaire based on the interviews and to finalize the questionnaire. Then, exploratory factor analysis was carried out on valid questionnaires from 48 enterprises obtained from the pretest. The results show that the data passed the KMO sample measure and Bartlett’s spherical test, the factor loadings were greater than 0.5, and the five-factor model fit index reached the standard. Hence, the scale had a certain degree of reliability and validity.

We collected formal survey data for the Jilin, Shandong, and Guangdong provinces and Beijing in China. We selected companies that achieved milestones in their digitalization, typically characterized by having configured and used digital technologies. The survey targets included senior managers (such as CEOs and presidents) and followers, with the assistance of their human resources departments. Questionnaires were distributed in three stages, in intervals of about 2 weeks, after which we used a back-translation process to create a Chinese version. In the first stage, employees filled in the questionnaire on digital leadership and provided basic personal information. In the second stage, leaders completed the questionnaires on digital entrepreneurial orientation, digital organizational culture, and big data analytics capabilities. In the third stage, leaders finished the questionnaire on exploratory innovation. To avoid response bias, the names of the measures were hidden, and the survey was anonymous. A total of 124 leaders and 586 employees from 124 enterprises participated in the survey. After excluding abnormal questionnaires, such as fill in the interruption, less than three followers per company, or regularity filling. In the end, 401 valid employee questionnaires and 88 valid leader questionnaires were obtained. The effective questionnaire recovery rate was 68%.

### Method

The variables involved in this paper are measurable by well-established scales, and the research questions are suitable for analysis and testing using large-sample empirical methods. In terms of research methodology, we analyzed the data using SPSS 24.0 and Mplus 7.4. Our hypotheses were tested using structural equation modeling and bootstrapping, which is more suitable for our research needs (McAlister, 2016). Therefore, we have chosen the appropriate approach for the problem to be studied.

### Measures and variables

We based on a seven-point Likert-type scale (from 1 = “strongly disagree” to 7 = “strongly agree”) to rate all items.

Digital leadership was measured using the scale developed by Zhu (2015) containing five dimensions: creativity, thinking and inquisition, curiosity, deep knowledge, and global vision and collaboration, which has been used several times in empirical studies. The scale has many items, we combined similar items to get a scale of 17 items [*α* = 0.986, CR = 0.987, AVE = 0.814; Average Rwg(j) = 0.929; ICC(1) = 0.317; ICC(2) = 0.678].

The innovativeness, proactiveness, and risk-taking dimensions of the 9-item digital entrepreneurial orientation scale was borrowed and adapted from Miller (1983) and Covin and Slevin (1991). We deleted a controversial item of product and service change nature (big change vs. minor change), and actively reformed the items. For the agility dimension, we used Lu and Ramamurthy’s (2011) 3-item scale on market agility. We used a total of 11 digital entrepreneurial orientation items (*α* = 0.942, CR = 0.944, AVE = 0.608).

We used 4 items from Martínez-Caro et al. (2020) to evaluate digital organizational culture (*α* = 0.866, CR = 0.870, AVE = 0.629).

Drawing on and improving upon the scale of Zhou and Wu (2010) and referring to the research design of Tuncdogan et al. (2017) on exploratory innovation, we selected and designed items by identifying commonalities between the two scales, for a total of six items (*α* = 0.922, CR = 0.924, AVE = 0.670).

Big data analytics capabilities were assessed according to 25 items by adopting the measures from Mikalef et al. (2019), which contains three dimensions: tangible resources, intangible resources, and human skills and knowledge. The scale has many items, we eliminated the items with factor load less than 0.6 and keep 22 items (*α* = 0.971, CR = 0.973, AVE = 0.628).

In this study, the ages of leaders, employees, and firms were used as control variables. This is because leaders’ age affects their work attitudes, values, and management style (Hambrick and Mason, 1984). Meanwhile, firms’ age has an impact on innovation (Coad et al., 2016). Younger employees and older employees do not respond to leader behavior and are not as motivated or capable of accepting digital change (Li et al., 2021). Actual age was used for leaders and employees. Firms’ age is measured by the natural logarithm of its established years.

Among these valid sample data, the youngest leader is 34 years old, and the oldest is 63 years old, with a mean of 49.92 and a standard deviation of 7.205. The youngest employee is 29 years old, and the oldest is 49 years old, with a mean of 35.921 and a standard deviation of 7.206. Most of the enterprises are 16–30 years old, accounting for 35.2%. From the perspective of industrial distribution, electronics, electrical, and information service industries are the main industries, accounting for 52.227%. The business involves electronic products, electronic components, household appliances and other product processing, sales, technology development, and technical services based on computer and communication network technology. Followed by the food, beverage, and medical industries, accounting for 20.454%. The offering is mainly wholesale, retail, and processing of diverse food, beverage and medical.

## Results

### Common method bias

To avoid common method bias, this study not only collected the questionnaire in stages but also used the dual data source method of leader–employee matching while conducting Harman’s one-way test of the data. The results show that the five factors explained a total of 72.842% of the total variance and the unrotated first factor explained 35.237% of the total variance of the variables, which did not exceed 40%, and the validated factor analysis shows that the five-factor model significantly outperformed the other models, so the variables in this study did not produce serious common method bias.

### Means and correlations

The results of the descriptive statistical analysis of the means, standard deviations, and correlation coefficients of each variable obtained using SPSS23.0 software are shown in Table 1. We found that the core variables significantly correlated with each other, and the correlation coefficients were all less than 0.7. Thus, we found the correlation between the core variables was basically consistent with the hypothesis.

**Table 1:** Means, SD, and correlations of all variables.

Variables	1	2	3	4	5	6	7	8
1 Leaders’ age								
2 Employees’ age	0.213*							
3 Firms’ age	0.376**	0.024						
4 DL	−0.176	−0.096	−0.121					
5 DEO	−0.295*	−0.203	−0.073	0.487**				
6 DOC	−0.133	−0.015	−0.268**	0.386**	0.324**			
7 EI	−0.042	−0.136	−0.025	0.538**	0.496**	0.479**		
8 BDAC	−0.262*	−0.098	0.037	0.214*	0.204	0.074	0.220*	
M	49.920	35.921	2.500	5.046	5.349	5.136	5.130	5.320
SD	7.205	7.206	0.894	0.705	0.542	0.588	0.642	0.647

DL, digital leadership; DEO, digital entrepreneurial orientation; DOC, digital organizational culture; EI, exploratory innovation; BDAC, data analytics capabilities. **p<0.01; *p<0.05.

### Measurement model

We used a large number of items to measure digital leadership, digital entrepreneurial orientation, and big data analytics capabilities. To prevent inflated measurement errors in latent variables caused by multiple items, according to Landis et al. (2000), we retained one question item per dimension for packaging. After packaging, digital leadership comprised five measurement items, digital entrepreneurial orientation comprised four measurement items, and big data analytics capabilities comprised three measurement items.

The lowest standardized factor loading value for each scale was over 0.6, the lowest CR value was over 0.7, and the lowest AVE value was over 0.5. The scales that fit the content and were widely cited were selected, revised, and improved several times to ensure content validity. The results of the discriminant validity of the variables are shown in Table 2. We found that the five-factor model fit the criteria and was the best. Further, we performed the HTMT test to verify the discriminant validity. The HTMT test requires the calculation of a ratio of the average correlations between constructs to the geometric mean of the average correlations within items of the same constructs (Voorhees et al., 2016). The results demonstrate that the highest HTMT value between the two factors is 0.564, which is less than 0.8. It can be judged that the sample data has good discriminant validity.

**Table 2:** Alternative model test results for the study variables.

Model	*χ* ^2^	*df*	RMSEA	CFI	TFI	SRMR
Five-factor	291.898	199	0.072	0.960	0.953	0.055
Four-factor	562.223	203	0.142	0.839	0.817	0.123
Three-factor	829.399	206	0.185	0.720	0.687	0.161
Two-factor	972.411	208	0.204	0.657	0.619	0.176
One-factor	1317.674	209	0.246	0.503	0.450	0.197

Five-factor model: digital leadership, digital entrepreneurial orientation, digital organizational culture, exploratory innovation, and big data analytics capabilities; four-factor model: based on the five-factor model, with digital leadership and exploratory innovation combined into one factor; three-factor model: combining digital leadership, exploratory innovation, and digital entrepreneurship orientation into one factor; two-factor model: digital leadership, exploratory innovation, digital entrepreneurial orientation, and digital organizational culture combined into one factor; one-factor model: all items load on a single factor.

### Structural model

The results of path analysis show that the path coefficient of digital leadership on digital entrepreneurial orientation was 0.401 (*p* = 0.000), on digital organizational culture was 0.327 (*p* < 0.001), on exploratory innovation was 0.343 (*p* = 0.006), on exploratory innovation was 0.394 (*p* < 0.001), and on exploratory innovation was 0.309 (*p* = 0.047).

Next, we tested the hypotheses by analyzing the main, mediating, and total effects among the variables in the theoretical model using bootstrapping (5,000 samples). According to the results of the data test presented in Table 3, we found that the relationship between digital leadership and exploratory innovation was significant (*B* = 0.309, *p* = 0.047, 95% CI: 0.034, 0.550) Therefore, H1 was fully supported. The indirect effect through digital entrepreneurial orientation was significant (*B* = 0.138, *p* < 0.001, 95% CI: 0.084, 0.171), as was the indirect effect through digital organizational culture (*B* = 0.129, *p* < 0.01, 95% CI: 0.111, 0.205). As such, H2 and H3 were supported. Furthermore, both the total mediating effect of digital leadership on exploratory innovation achieved through multiple mediators (*B* = 0.266, *p* < 0.01, 95% CI: 0.137, 0.342) was significant.

**Table 3:** Bootstrapping effect analysis.

	*B*	Coefficient		Bootstrapped 95% CI	
		SE	*p*-value	LL	UL
**Direct effect**					
Direct	0.309	0.155	0.047	0.034	0.550
**Indirect effect**					
Mediate 1	0.137	0.039	0.000	0.085	0.172
Mediate 2	0.129	0.045	0.004	0.111	0.205
Total mediate	0.266	0.077	0.001	0.137	0.342

*Note*: Mediate 1 represents the indirect effect through digital entrepreneurial orientation; mediate 2 represents the indirect effect through digital organizational culture.

We further used the latent moderated structure (LMS) model to construct a structural equation model with digital entrepreneurship orientation, big data analytics capability, the interaction term as the independent variable, and exploratory innovation as the dependent variable. The results showed that the effects of digital entrepreneurial orientation (*B* = 0.397, *p* < 0.05), big data analytics capabilities (*B* = 0.275, *p* < 0.05), and the interaction term between the two (*B* = 0.474, *p* < 0.050) on exploratory innovation were significant. Hence, H4 was supported.

We conducted the same test to examine the moderating role of big data analytics capabilities between digital organizational culture and exploratory innovation. The results show that the effect of digital organizational culture (*B* = 0.406, *p* < 0.001) and the interaction term between digital organizational culture and big data analytics capabilities (*B* = 0.346, *p* < 0.05) on exploratory innovation were both significant. As a result, H5 was supported.

**Figure 3:**
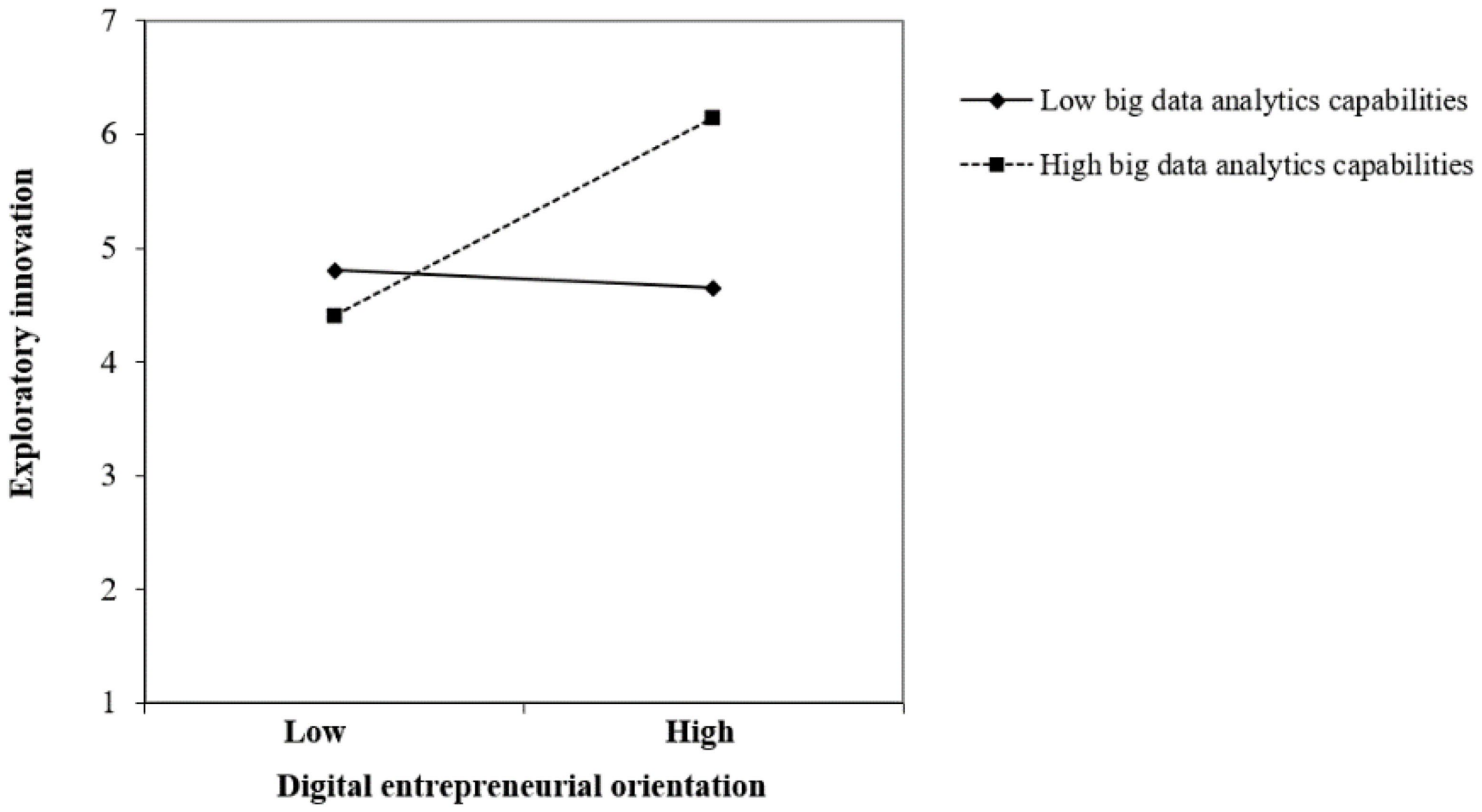
The moderating effect of big data analytic capabilities on the relationship between digital entrepreneurship orientation and exploratory innovation.

**Figure 4:**
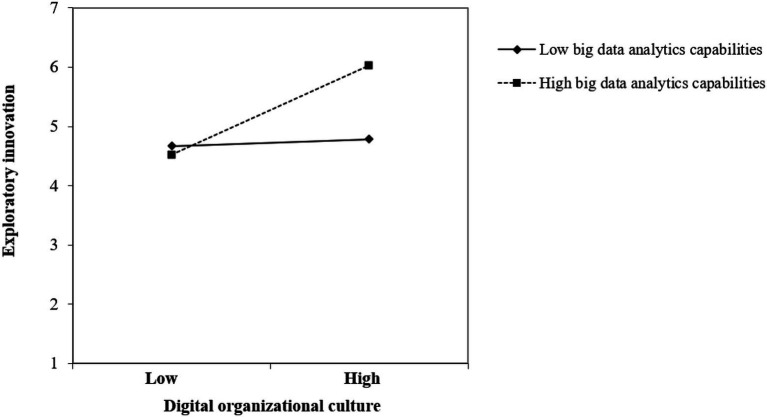
The moderating effect of big data analytic capabilities on the relationship between digital organizational culture and exploratory innovation.

We further display the moderating effect of big data analytics capabilities on the relationship between digital entrepreneurship orientation and exploratory innovation in Figure 3 as well as that between digital organizational culture and exploratory innovation in Figure 4.

### Moderated mediation model test

On this basis, one standard deviation was added or subtracted from the mean of the moderating variable big data analysis capabilities, so as to distinguish the mediating effect of digital entrepreneurship orientation and digital organizational culture between the independent variable digital leadership and the dependent variable exploratory innovation under different levels of big data analysis ability. The test results are given in Table 4. For the mediating variable of digital entrepreneurial orientation, when it is at a low level, the boot 95%CI includes the number 0, which means that there is no mediation effect at this level; when it is at the average level, the boot 95%CI does not include the number 0, it means that there is a mediating effect at this level, and the effect value is 0.094; when it is at a high level, the boot 95%CI does not include the number 0, which means that there is a mediating effect at this level, and the effect value is 0.181. The analysis is shown that at different levels, the mediating effect is inconsistent, indicating that there is a mediating effect, and H6 is established. In the same way, H7 is established.

**Table 4:** Moderated mediation test.

Mediator	Condition	*b*	SE	Bootstrap 95% CI
Digital entrepreneurial orientation	Low	0.007	0.070	−0.088, 0.190
	Middle	0.094	0.052	0.009, 0.217
	High	0.181	0.076	0.048, 0.348
Digital organizational culture	Low (−1SD)	0.056	0.044	−0.014, 0.161
	Middle	0.088	0.039	0.027, 0.179
	High (+1SD)	0.120	0.050	0.038, 0.235

## Discussion and conclusions

Leadership has evolved over the last few decades. Modern leadership focus not only on the leader, but also on the followers, the work environment, and the culture. Today, with digitalization sweeping the globe, leadership is required to evolve to become collective, relational, strategic, global, and dynamic (Larjovuori et al., 2018). The task of a leader is not intended to rule, but to accompany, participate and listen (Schwarzmüller et al., 2018). Researchers are working to examine the definition and characteristics of digital leadership. Scholars have developed different conclusions and generally agree that digital leadership emphasizes five vital capabilities for leaders: creativity, thinking and inquisition, curiosity, deep knowledge, and global vision and collaboration (Zhu, 2015). The competitive environment is increasing in complexity, volatility, unpredictability, and speed of change (Schiuma et al., 2021). Organizations have to face unexpected, unclear scenarios and unpredictable challenges. They must boldly transform their businesses and behaviors to turn challenges into development and growth opportunities. How leaders lead their organizations to achieve exploratory innovation in the digital age has grown up to be a critical issue. However, to the best of our knowledge, previous studies have not explored the relationship between digital leadership and exploratory innovation. Therefore, this study attempts to address this unknown issue in the literature and investigate it empirically. In particular, we have utilized RBV and SIP, using time-lagged survey data from 401 followers and 88 leaders, to explore how digital leadership affects digital entrepreneurial orientation, digital organizational culture, and ultimately exploratory innovation. Our study provides detailed information about digital leadership combining business and IT strategies to form a digital business strategy that values exploratory innovation as a fundamental element and supports it with initiatives. In addition, digital leadership encourages the diffusion and application of digital tools within the enterprise and promotes positive human-machine interactions such that employees have the time, motivation, and ability to engage in more creative work and consider exploratory opportunities.

One of the innovative contributions of this study is to explore the mediating role of digital entrepreneurial orientation and digital organizational culture between digital leadership and exploratory innovation. Mihardjo et al. (2019a) found that digital leadership significantly influenced corporate innovation management but failed to show the specific pathways and mechanisms. To advance the research towards depth, mediating variables need to be explored from appropriate perspectives. To this end, we drew upon the ideas of Oberer and Erkollar (2018) and Nadkarni and Prügl (2021) on the division of the actor and material aspects of digitalization research. This article concludes that digital entrepreneurial orientation and digital organizational culture play mediating roles in digital leadership and exploratory innovation based on the perspectives of RBV and SIP. Specifically, considering RBV, digital leadership provides the resources needed to develop digital entrepreneurial orientation. Such digital entrepreneurial orientation is a unique resource that motivates companies to reinforce exploratory innovation without being afraid of taking risks when facing potential opportunities. From the view of SIP, digital leadership provides valued environment clues to followers. Digital leadership communicates digital vision and information with followers and grants them the right, trust, tolerance, and respect. Members are motivated to take an active part in building a unified digital organizational culture. Digital technologies can be fully exploited to promote knowledge sharing and creation when the digital organizational culture matches the values of digital technologies. Thereby positively influencing exploratory innovation. It is worth noting that digital entrepreneurial orientation and digital organizational culture have also not been discussed together with digital leadership. That is, this paper is also innovative in its exploration of digital leadership as it affects digital entrepreneurial orientation and digital organizational culture.

This study also provides new findings on the relationship between big data analytics capabilities in digital entrepreneurial orientation and exploratory innovation, as well as the relationship between digital organizational culture and exploratory innovation. Specifically, the findings suggest that according to the theory of RBV and dynamic capability, companies with a digital entrepreneurial orientation have stronger big data analytics capabilities. So more valuable information can be generated. Big data analytics capabilities support more proactively and quickly identifying and responding to upcoming opportunities such that enterprises are more likely to develop new products, new technologies, and new production processes associated with a higher degree of innovation and complexity. In companies with digital organizational culture, the stronger the big data analytics capabilities, the more companies are likely to make informed decisions and drive collaboration among decentralized organizational units to facilitate exploratory innovation. Further, big data analysis capabilities have the positive moderating effect on the mediating effect of digital entrepreneurship orientation and digital organizational culture, that is, when enterprises show a high level of big data analysis capabilities, the above mediating variables have a strong mediating effect between digital leadership and exploratory innovation.

In summary, this study provides unique insights by investigating the mediating role of digital entrepreneurial orientation and digital organizational culture in the impact of digital leadership on exploratory innovation. The results suggest that digital leadership can influence exploratory innovation through the digital organizational culture of organizational management and also through the digital entrepreneurial orientation of business management, which is enhanced by big data analytics capabilities. This study provides interesting and valuable guidance for researchers interested in understanding the impact of digital leadership on exploratory innovation.

### Theoretical implications

Our findings make several important contributions to present knowledge. First, the existing research on digitalization has primarily focused on the discussion of products, services, models, and processes, while the digitalization of organizations has been underestimated (Cortellazzo et al., 2019). As a result, the study on the digitalization of organizations has just ignited, with a small and very fragmented existing literature scattered across such topics as digital leadership styles, changes in employee work styles, use of technology, performance and talent management, and organizational systems (Schwarzmüller et al., 2018). The depth and breadth of the research questions are insufficient, and there is imperative to explore the digital impact on leaders and followers (Bharadwaj et al., 2013; Schiuma et al., 2021). This study delves into the digitalization of organizational domains to discuss the role of “people.” Specifically, it explores the role of digital leadership in digitalization and develop a theoretical model of digital leadership influencing exploratory innovation behavior. We enrich digitalization and digital leadership research, responding forcefully to scholars such as Hesse (2018), who call for strengthening research on the digitalization of organizations.

Second, although leadership is one of the most researched areas in organizational science, digitalization is a rather young and unexplored phenomenon. Scholars such as Zhu (2015), Waal et al. (2016), and McCarthy et al. (2021) have developed richer accounts of the characteristics or capabilities of digital leadership, but the impact outcomes of digital leadership are limited to dynamic capability (Mihardjo et al., 2019a), strategic alliances, market orientation (Mihardjo et al., 2019b), innovation management (Wasono and Furinto, 2018). What else digital leadership can deliver has not been fully discussed (Hesse, 2018). In particular, in the study of the relationship between digital leadership and exploratory innovation, the existing literature only recognizes that there is a positive relationship between the two (Mihardjo et al., 2019a), but the involved impact pathways and mechanisms are still not being sufficiently discussed. Accordingly, using the RBV and SIP, we more comprehensively analyzed the mechanisms and pathways by which digital leadership influences exploratory innovation according to two perspectives: strategic orientation and organizational culture, with digital entrepreneurial orientation and digital organizational culture as mediating variables. And based on RBV and dynamic capability theory, the moderating role of big data analysis capability is explained. Obviously, this article is beneficial to comprehend the role of digital leadership in innovation.

Third, previous antecedent leadership style variables for exploratory innovation have mainly included traditional leadership styles such as transformational leadership (Chen et al., 2019), inclusive leadership (Gong et al., 2021), participative leadership (Chang et al., 2019), and distributed leadership (Berraies et al., 2021). Among the various factors that may influence exploratory innovation, digital leadership has emerged as a new focus of attention. This study indicates the importance of a new type of leadership, namely digital leadership, on exploratory innovation in the context of digitalization, the greatest reality facing companies today. We contribute a valuable addition to the research on what leadership styles leaders adopt to advance exploratory innovation and deepen the understanding of the formation mechanism of exploratory innovation.

Finally, the gradual emergence of strategic orientation in digitalization research has pushed us to consider how entrepreneurial orientation works in digital contexts and what its new manifestations are. However, existing studies have not thought about this in depth and have mostly used entrepreneurial orientation variables directly from traditional contexts (Ritala et al., 2021). Our study confirms that digital products or services are changing significantly faster in the digital era and are intermittent and cyclical, requiring an agile and lean approach (Lu and Ramamurthy, 2011). The finding adds a new market agility dimension to the original dimensions of entrepreneurial orientation such as innovativeness, proactiveness, and risk taking to form a new variable of digital entrepreneurial orientation.

### Practical implications

Our findings offer several implications for leveraging digital leadership to strengthen exploratory innovation.

First, our findings show that digital leadership positively impacts exploratory innovation, which revealed that leaders should be fully aware of the importance of digital leadership and make efforts to improve their digital literacy and leadership skills in numerous ways, such as through consciously learning and actively participating in related training courses and forums (Besson and Rowe, 2012; Borowska, 2019). Corporate boards can hire digital leaders with creativity, thinking and inquisition, curiosity, deep knowledge, and global vision and collaboration during the selection or recruitment process (Zhu, 2015; Borowska, 2019) to fulfilling the potential of digital leadership in steering the company to continuously create new value and compete in the digital economy era.

Second, the findings indicate that digital entrepreneurial orientation and digital organizational culture play mediating roles between digital leadership and exploratory innovation, which showed that when companies engage in exploratory innovation, digital leadership should be given more attention. Digital leadership is required to support exploratory innovation from the digital business strategic level down to the specific execution level (Bharadwaj et al., 2013) and reasonably internalize digital entrepreneurial orientation into corporate behavioral rules. In addition, attention should be paid to the formation of digital organizational culture. Members must be supported in improving digital knowledge and literacy through training and communication, increasing their participation and initiative through performance rewards and establishing upward channels (Naqshbandi and Tabche, 2018). Hence, exploratory innovation can be rapidly developed.

Third, the results show that big data analytics capabilities positively moderate the relationship between digital entrepreneurial orientation and exploratory innovation and between digital organizational culture and exploratory innovation, which shows companies that it is insufficient to only buy big data analytics equipment and tools: they must also build related capabilities. They should invest in the tangible and intangible resources and human skills areas required to achieve big data analytics capabilities in the long term (Mikalef et al., 2017). Specifically, digital infrastructure should be continuously updated, IT departments should be encouraged to experiment with advanced analytical technology, people with excellent technical and managerial understanding of big data should be recruited, and then big data analytics insights should be incorporated into organizational operations and decision making (Su et al., 2021).

### Limitations and future research directions

Although our findings contribute to the research on and practice of digital leadership in impacting exploratory innovation, this research still has shortcomings. First, regarding sample collection, the sample in this study was taken from Chinese enterprises. It is unclear what extent these findings are applicable to companies in other countries and regions. Future research should collect data from a wider range of countries and regions and from a wider variety of industries, and further compare and analyze the findings with those of this study to verify their generalizability.

Second, in this study, we did not control for other positive leadership styles, such as transformational leadership and moral leadership. Future research should control for the impact of similar leadership styles on exploratory innovation to enhance the robustness of the results.

Third, in terms of data processing, we conducted a two-stage time-lagged study in which questionnaires were filled out by both leaders and employees, which may lessen transient response biases and common method biases. Future research could use longitudinal empirical studies or case studies to confirm causality in the model.

Fourth, the mechanisms of digital leadership based on other perspectives to influence exploratory innovation need to be further explored. Depending on the RBV and SIP, this study explores the pathways and mechanisms between digital leadership and exploratory innovation from the two perspectives of strategic orientation and organizational culture, but it does not deny that there may be other divisional perspectives in finding the mediating variables between digital leadership and exploratory innovation as well as other path mechanisms. Therefore, future research should explore these mechanisms from different perspectives, such as the leader–member relationship, organizational climate, and organizational system.

Fifth, future research should continue to explore the antecedent and outcome variables of digital leadership, thereby expanding the framework. Digital leadership is an emerging and promising research area. In the existing literature, digital leadership focuses on characteristics and competencies (McCarthy et al., 2021), while influencing factors, formation processes, impact outcomes, and mechanisms of action have not been fully discussed, resulting in existing studies failing to answer questions such as how to establish digital leadership and how digital leadership works. Future research should discuss the influencing factors and formation process of digital leadership in terms of organizational factors (e.g., corporate characteristics, TMT team characteristics), environmental factors (e.g., competitive pressure, market demand), and technological factors (e.g., digital infrastructure, digital capabilities). The impact outcomes and mechanisms of digital leadership can be explored at the employee level (e.g., employee transgressive innovation behavior), the leader–employee level (e.g., leader empowerment and trust of employees), and the organizational level (e.g., organizational agility).

Finally, this study develops the concept and dimensions of digital entrepreneurial orientation, but it is only the beginning of digital entrepreneurial orientation research. Future studies can consider continuing to enrich the research around the formation mechanisms and impact outcomes of digital entrepreneurship orientation in other digital scenarios (e.g., digital transformation, digital innovation ecosystem, digital entrepreneurship ecosystem).

## Data availability statement

The original contributions presented in the study are included in the article, further inquiries can be directed to the corresponding authors.

## Author contributions

TW and XL were responsible for the data collection of the article and the writing of the article. FS was responsible for the questionnaire design of the article and providing revised advice. All authors contributed to the article and approved the submitted version.

## Funding

This work was supported in part by the Philosophy and Social Science Foundation of Heilongjiang Province (21GLC190, 20GLC206); Heilongjiang Key Research Projects for Economic and Social Development (21216); the National Office of Philosophy and Social Sciences of China (21FYB064); and the Fundamental research Funds for the central universities of China (3072021CFJ0905).

## Conflict of interest

The authors declare that the research was conducted in the absence of any commercial or financial relationships that could be construed as a potential conflict of interest.

## Publisher’s note

All claims expressed in this article are solely those of the authors and do not necessarily represent those of their affiliated organizations, or those of the publisher, the editors and the reviewers. Any product that may be evaluated in this article, or claim that may be made by its manufacturer, is not guaranteed or endorsed by the publisher.

## Appendix

### Questionnaire: Digital leadership

**Table:**

Dimension	Items	Factor loading
Creativity	CR1. The top leader of our company is creative businesses leader with though ability	0.913
	CR2. The top leader of our company has creativity and innovative mindset	0.900
	CR3. The top leader of our company could formulate the idea of the future into reality of business	0.873
	CR4. The top leader of our company constantly seeking change	0.871
Thinking and inquisition	TI1. The top leader of our company always reflects	0.924
	TI2. The top leader of our company continues to explore problems at work	0.903
	TI3. The top leader of our company has profound knowledge, ability, and depth of understanding	0.901
Curiosity	CU1. The top leader of our company keeps his/her thirst for knowledge to learn and adapt to change	0.908
	CU2. The top leader of our company has the learning capability	0.920
	CU3. The top leader of our company has the capability to implementing the learning and digital capability	0.885
Deep knowledge	DK1. The top leader of our company masters the trend of scientific and technological development	0.879
	DK2. The top leader of our company is proficient in digital technology	0.902
	DK3. The top leader of our company has the knowledge and understand in depth in terms of policy	0.923
	DK4. The top leader of our company by using their interpretation, assumption and synthesizing the information could profound the knowledge to take the decision making.	0.914
Global vision and collaboration	GVC1. The top leader of our company has a global vision and vision	0.918
	GVC2. The top leader of our company has the ability to provide direction and could become an orchestra in transforming the digital business transformation	0.913
	GVC3. The top leader of our company actively builds strong domestic and global networks	0.888

All factor loading results are supported by Mplus7.4, the same as the following tables; *α* = 0.986, CR = 0.987, AVE = 0.814; This scale refers to Zhu (2015).

### Digital entrepreneurial orientation

**Table:**

Dimension	Items	Factor loading
Innovativeness	I1. In general, our company favors a strong emphasis on the marketing and innovation of tried-and-true products or services	0.707
	I2. In the past 3 years, our company has launched and sold a series of new products or services	0.845
Proactiveness	P1. In dealing with its competitors, we typically respond to actions which competitors initiate	0.739
	P2. In dealing with its competitors, we always the first business to introduce new products/services, administrative techniques, operating technologies, etc.	0.789
	P3. In dealing with its competitors, we usually take action first, and then competitors follow up or respond	0.810
Risk-taking	RT1. We have a strong proclivity for high-risk projects (with normal and certain rates of return)	0.757
	RT2. We tend to take bold and quick actions to achieve their goals.	0.838
	RT3. When confronted with decision-making situations involving uncertainty, we tend to take a bold and positive attitude and seize potential opportunities	0.778
Agility	A1. We are quick and implement appropriate decisions in the face of market/customer changes	0.721
	A2. We constantly look for ways to reinvent/reengineer our organization to better serve our market place	0.861
	A3. We treat market-related changes and apparent chaos as opportunities to capitalize quickly	0.717

*α* = 0.942, CR = 0.944, AVE = 0.608; This scale refers to Miller (1983), Covin and Slevin (1991), Lu and Ramamurthy (2011).

### Digital organizational culture

**Table:**

Items	Factor loading
DOC1. The teams collaborate functionally in the initiatives for innovation and digital transformation.	0.737
DOC2. There is a clear orientation to digital technology changes inside the organization’s culture.	0.708
DOC3. The culture of digital innovation and change takes part as a natural process within the organization.	0.811
DOC4. The organization share with the staff the digital strategy, taking into consideration their suggestions.	0.903

*α* = 0.866, CR = 0.870, AVE = 0.629; This scale refers to Martínez-Caro et al. (2020).

### Exploratory innovation

**Table:**

Items	Factor loading
EI1. We invent products and services for new markets	0.849
EI2. We experiment with new products and services in our markets	0.825
EI3. We experiment with products and services that are completely new to our company	0.834
EI4. We make breakthroughs in areas where we have no previous experience	0.840
EI5. We develop brand-new technologies and skills	0.813
EI6. We introduce completely improved products	0.748

*α* = 0.922, CR = 0.924, AVE = 0.670; This scale refers to Zhou et al. (2010), Tuncdogan et al. (2017).

### Big data analytics capabilities

**Table:**

Dimension	Items	Factor loading
Tangible resources	TR1. We have access to very large, unstructured, or fast-moving data for analysis	0.867
	TR2. We integrate data from multiple sources into a data warehouse for easy access	0.831
	TR3. We integrate external data with internal to facilitate analysis of business environment	0.754
	TR4.Our ‘big data analytics’ projects are adequately funded	0.721
	TR5.Our ‘big data analytics’ projects are given enough time to achieve their objectives	0.768
	TR6. We have explored or adopted parallel computing approaches to big data processing	0.709
	TR7. We have explored or adopted different data visualization tools	0.867
	TR8. We have explored or adopted cloud-based services for processing data and performing analytics	0.793
Intangible resources	IR1. We base our decisions on data rather than on instinct	0.799
	IR2. We are willing to override our own intuition when data contradict our viewpoints	0.762
	IR3. We continuously coach our employees to make decisions based on data	0.684
	IR4. We are able to acquire new and relevant knowledge	0.822
	IR5. We have made concerted efforts for the exploitation of existing competencies and exploration of new knowledge	0.827
	IR6. We are able to assimilate relevant knowledge	0.751
	IR7. We are able to apply relevant knowledge	0.836
Human skills and knowledge	HSK1. Our BDA managers are able to understand the business need of other functional managers, suppliers, and customers to determine opportunities that big data might bring to our business.	0.856
	HSK2. Our BDA managers are able to coordinate big data-related activities in ways that support other functional managers, suppliers, and customers	0.768
	HSK3. Our BDA managers are able to understand and evaluate the output extracted from big data	0.830
	HSK4. Our BDA managers are able to understand where to apple big data	0.817
	HSK5. Our ‘big data analytics’ staff is well trained	0.815
	HSK6. We provide big data analytics training to our own employees	0.749
	HSK7. Our ‘big data analytics’ staff has suitable education to fulfil their jobs	0.781

*α* = 0.971, CR = 0.973, AVE = 0.628; This scale refers to Mikalef et al. (2019).
